# A sensitive short read homology search tool for paired-end read sequencing data

**DOI:** 10.1186/s12859-017-1826-2

**Published:** 2017-10-16

**Authors:** Prapaporn Techa-Angkoon, Yanni Sun, Jikai Lei

**Affiliations:** Department of Computer Science and Engineering, Michigan State University, East Lansing, 48824 MI USA

**Keywords:** Short read homology search, Profile homology search, Profile HMM, Paired-end read alignment

## Abstract

**Background:**

Homology search is still a significant step in functional analysis for genomic data. Profile Hidden Markov Model-based homology search has been widely used in protein domain analysis in many different species. In particular, with the fast accumulation of transcriptomic data of non-model species and metagenomic data, profile homology search is widely adopted in integrated pipelines for functional analysis. While the state-of-the-art tool HMMER has achieved high sensitivity and accuracy in domain annotation, the sensitivity of HMMER on short reads declines rapidly. The low sensitivity on short read homology search can lead to inaccurate domain composition and abundance computation. Our experimental results showed that half of the reads were missed by HMMER for a RNA-Seq dataset. Thus, there is a need for better methods to improve the homology search performance for short reads.

**Results:**

We introduce a profile homology search tool named Short-Pair that is designed for short paired-end reads. By using an approximate Bayesian approach employing distribution of fragment lengths and alignment scores, Short-Pair can retrieve the missing end and determine true domains. In particular, Short-Pair increases the accuracy in aligning short reads that are part of remote homologs. We applied Short-Pair to a RNA-Seq dataset and a metagenomic dataset and quantified its sensitivity and accuracy on homology search. The experimental results show that Short-Pair can achieve better overall performance than the state-of-the-art methodology of profile homology search.

**Conclusions:**

Short-Pair is best used for next-generation sequencing (NGS) data that lack reference genomes. It provides a complementary paired-end read homology search tool to HMMER. The source code is freely available at https://sourceforge.net/projects/short-pair/.

## Background

Homology search has been one of the most widely used methods for inferring the structure and function of newly sequenced data. For example, the state-of-the-art profile homology search tool, HMMER [1] has been successfully applied for genome-scale domain annotation. The major homology search tools were designed for long sequences, including genomic contigs, near-complete genes, or long reads produced by conventional sequencing technologies. They are not optimized for data produced by next-generation sequencing (NGS) platforms. For reads produced by pyrosequencing or more recent PacBio and nanopore technologies, frameshift caused by sequencing errors are the major challenges for homology search. For data sets produced by Illumina, short reads will lead to marginal alignment scores and thus many reads could be missed by conventional homology search tools. In order to apply homology search effectively to NGS data produced by Illumina, many of which contain short reads, read mapping or *de novo* assembly [2–6] is first employed to assemble short reads into contigs. Then existing homology search tools can be applied to the contigs to infer functions or structures.

However, it is not always feasible to obtain assembled contigs from short reads. For example, complex metagenomic data poses serious computational challenges for assembly. Just 1 gram of soil can contain 4 petabase pairs (1×10^15^ bps) of DNA [7] and tens of thousands of species. Read mapping is not very useful in finding the native genomes or genes of these reads as most reference genomes are not available. *De novo* assembly also has limited success due to the complexities and large sizes of these data [4, 5, 8]. Besides metagenomic data, which usually lack complete reference genomes, RNA-Seq data of non-model species also faces similar computational challenges. Assembling short reads into correct transcripts without using any reference genome is computationally difficult.

Thus, in order to analyze the NGS data without reference genomes, a widely adopted method for functional analysis is to classify reads into characterized functional classes, such as protein/domain families in Pfam [9, 10], TIGRFAM [11], FIGfams [12], InterProScan [13], FOAM [14], etc. The read assignment is usually conducted by sequence homology search that compares reads with reference sequences or profiles, i.e., a family of homologous reference sequences. The representative tools for sequence homology search and profile homology search are BLAST [15] and HMMER [1], respectively. Profile homology search has several advantages over pairwise alignment tools such as BLAST. First, the number of gene families is significantly smaller than the number of sequences, rendering much faster search time. For example, there are only about 13,000 manually curated protein families in Pfam, but these cover nearly 80% of the UniProt Knowledgebase and the coverage is increasing every year as enough information becomes available to form new families [10]. The newest version of HMMER [1] is more sensitive than BLAST and is about 10% faster. Second, previous work [16] has demonstrated that using family information can improve the sensitivity of a remote protein homology search, which is very important for metagenomic analysis because many datasets contain species remotely related to ones in the reference database.

**Fig. 1:**
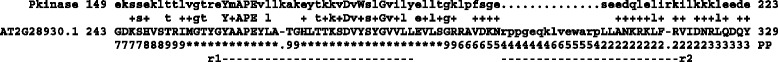
An example of a protein family, its alignment with a gene, and read mapping positions of a read pair against the gene. The Pkinase model had annotation line of consensus structure. The line beginning with Pkinase is the consensus of the query model. Capital letters show positions of the most conservation. Dots (.) in this line represent insertions in the target gene sequence with respect to the model. The midline represents matches between the Pkinase model and the AT2G28930.1 gene sequence. A + represents positive score. The line beginning with AT2G28930.1 is the target gene sequence. Dashes (-) in this line represents deletions in the gene sequence with respect to the model. The bottom line indicates the posterior probability of each aligned residue. A 0 represents 0-5%, 1 represents 5-15%,..., 9 represents 85-95%, and * represents 95-100% posterior probability. The line starting with *r*_1_ and ending with *r*_2_ is read mapping regions on the gene sequence. A - indicates where the position of the read can be mapped to the gene sequence

HMMER has been successfully used in genome-scale protein domain annotation in many species. It has both high specificity and sensitivity in identifying domains. Thus, it is also widely adopted for profile homology search in a number of existing NGS analysis pipelines or websites (e.g. IMG/M [17], EBI metagenomics portal [18], CoMet [19], HMM-FRAME [20], SALT [21], SAT-Assembler [22], etc.). However, HMMER is not optimized for short-read homology searches. Short reads sequenced from regions of low conservation tend to be missed. One example is shown in Fig. 1, which revealed the short-read alignments using the whole gene alignment against the protein domain and the read mapping positions on the gene. In this example, one end *r*_1_ can be aligned to the domain using HMMER with filtration on. However, the other end *r*_2_ cannot be aligned by HMMER because of its poor conservation against the underlying protein family. In addition, we have quantified the performance of HMMER on several real NGS datasets. The results showed that HMMER has much lower sensitivity when it is applied to short reads than to complete genes or genomes.

In order to improve the sensitivity, one may consider to use loose cutoffs such as a low score or high E-value cutoff. However, using loose cutoffs can lead to false positive domain alignments. In this work, we will describe a new method to improve the sensitivity of profile homology search for short reads without jeopardizing the alignment accuracy. The implementation, named Short-Pair, can be used together with HMMER to increase the homology search performance for short reads.

## Methods

In this section, we describe a short read homology search method that incorporates properties of paired-end read sequencing. Paired-end sequencing is the preferred sequencing mode and is widely adopted by many sequencing projects. We have observed that for a large number of read pairs, only one end can be aligned by HMMER while the other end is missed. Thus, we exploit the sequencing property of paired-end reads to rescue the missing end.

Our probabilistic homology search model quantifies the significance of the alignment between a read pair and a protein domain family. The computation incorporates the distribution of fragment lengths (or insert sizes) of paired-end reads and the alignment scores. Similar approaches have been applied to mapping paired-end DNA reads to a reference genome [23, 24]. But to our knowledge, this is the first time that an approximate Bayesian approach has been employed to align paired-end reads to protein families.

There are three major steps. In the first step, we will align each end (all-frame translations) to given protein families using HMMER under E-value cutoff 10. Note that although GA-cutoff is the recommended cutoff by HMMER for accurate domain annotation, only a small percentage of short reads can pass GA cutoff. Thus, we use E-value cutoff 10 in the first step in order to recruit more reads. As the reads are short, this step will usually align each read to one or multiple protein families. Not all of the alignments are part of the ground truth. In the second step, for all read-pairs where only one end is aligned by HMMER, we use the most sensitive mode of HMMER to align the other end to the protein families identified in the first step. Although the sensitive search mode of HMMER is slow, it is only applied to the specified protein families that are substantially fewer than total protein families in the dataset and thus will not become the bottleneck of large-scale homology search. In the last step, the posterior probability of the alignment between a pair of reads and a protein domain family is calculated.

**Fig. 2:**
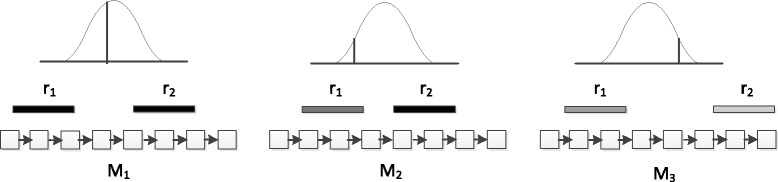
HMM alignments of a read pair. Paired-end reads *r*_1_ and *r*_2_ represented by two greyscale lines are aligned against models *M*_1_, *M*_2_, and *M*_3_ with different scores of alignments. The darker lines represent bigger scores. The fragment size distribution is provided above each model. The distance between the two alignments is computed and is used to compute the likelihood of the corresponding fragment size. In this example, *M*_1_ is most likely to be the native family

The falsely aligned domains in the first step will be removed in the last step through the computation of the posterior alignment probability. Figure 2 shows an example about determining the true protein family if both ends can be aligned to several families. In this example, *M*_1_ is the most likely to be the native family due to the bigger alignment scores and the higher probability of the observed fragment length. We quantify the posterior probability of each read pair being correctly aligned to a protein family.

As the example in Fig. 2 shows, in order to calculate the posterior probability of an alignment, we need to know the size distribution of fragments, from which paired-end reads are sequenced. Usually we may have the information about the range of the fragments (shortest and longest). However, the size distribution is unknown. For metagenomic data and RNA-Seq data of non-models species whose complete or quality reference genomes are not available, it is not trivial to derive the fragment size distribution. In this work, we take advantage of the protein alignment and the training sequences to estimate the fragment size distribution. The next two sections will describe the details about computing fragment size distribution and the method to rank alignments using posterior probabilities.

### Constructing fragment length distribution

Paired end reads are sequenced from the ends of fragments. When the reference genome is available, the fragment size can be computed using the distance between the mapping positions of the read pair. Thus, the distribution profile can be computed [23, 24] from a large-scale of read mapping positions. However, this method is not applicable to our work because we are focusing on the homology search of NGS data that lack reference genomes. For these data, we propose a model-based method to estimate fragment size distribution. The key observation is that if a read pair can be uniquely aligned to a protein family, it is very likely that this pair is sequenced from a gene that is homologous to the member sequences of the protein family. The homology is inferred from statistically significant sequence similarity. Thus, we will use the alignment positions and the homologous seed sequences to infer the fragment size. This method is not accurate as we are not using any reference genomes/genes. However, our experimental results have shown that the estimated distribution is very close to the true distribution.

**Fig. 3:**
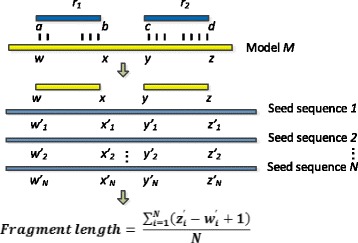
Calculating the fragment size for a read pair. The alignment positions along the profile HMM can be converted into positions in each seed sequences. The fragment size is computed as the average size of those mapped regions

Figure 3 sketches the main steps of inferring a fragment’s size from the alignment of a read pair against a protein family model. A read pair *r*_1_ and *r*_2_ are uniquely aligned to a protein family *M*. The alignment positions along the model *M* are from *w* to *x* and *y* to *z*, respectively. Model *M* is trained on a group of homologous sequences (“seed sequence 1” to “seed sequence N”). Note that the actual sequence from which *r*_1_ and *r*_2_ are sequenced is not in the training set of model *M*. The alignment positions along the model *M* will be first converted into the column indices in the multiple sequence alignment constructed by all seed sequences. Then after accounting for deletions and insertions, the column indices will be converted into positions along each seed sequence. As it is unknown which seed sequence shares the highest sequence similarity with the gene containing the fragment, we calculate the fragment size as the average of the distances between converted alignment positions.

Figure 3 only shows the fragment size estimation for one read pair. In order to construct the fragment size distribution, we use the fragment sizes computed for all paired-end reads that are uniquely aligned to protein domain families. As shown in Fig. 1, when both ends can be aligned uniquely to a protein family, usually these ends are sequenced from a region with high conservation. Thus, most of the estimations are close to the truth. However, for protein families or domains that contain many remote homologs, it is likely that the fragment size estimation is very different from the true fragment size. These wrong estimations either become outliers of the whole distribution or will slightly change the pattern of the fragment size distribution according to our experimental results. We will compare the inferred distribution with the ones that are derived based on read mapping results.

### Probabilistic model

For each aligned paired-end read, an approximate Bayesian approach [23, 24] is used to estimate the “alignment quality.” The quality of alignment is defined as the probability of a pair of reads being accurately aligned to its native protein domain family. Because a pair of reads could be aligned to multiple domain families and some of them might not be in ground truth, we can rank all alignments using computed posterior probabilities and keep the alignments with high probability.

Let *r*_1_ and *r*_2_ be a read pair. Let *A*_1_ and *A*_2_ be the candidate alignment sets of *r*_1_ and *r*_2_ against one or more protein family models. For each alignment pair *a*_1_ ∈ *A*_1_ and *a*_2_ ∈ *A*_2_ with *a*_1_ and *a*_2_ being aligned to the same protein family *M*, we calculate the posterior probability of *a*_1_ and *a*_2_ being the true alignments generated by the read pair *r*_1_,*r*_2_ against *M* as:

1 $$ Pr\left(a_{1}, a_{2}| r_{1}, r_{2}\right) \propto e^{s_{a_{1}}/T}e^{s_{a_{2}}/T}Pr\left(f_{r_{1}, r_{2}}\right)  $$

where $e^{s_{a_{1}}/T}$ is the target probability of generating an alignment score of *a*_1_ against *M* [1, 25]. *T* is the scaling factor used in E-value computation. $Pr(f_{r_{1}, r_{2}})$ is the probability of observed fragment size between *r*_1_ and *r*_2_. The posterior probability depends on the fragment length computed from *a*_1_ and *a*_2_ as well as their alignment scores.

We compute Eq. (1) for each read pair’s alignments and keep the alignments above a given threshold. For each read pair, suppose the maximum posterior probability of its alignments against all aligned models is *p*_*max*_. We keep all alignments with probabilities above *p*_*max*_×*τ*, where *τ* is 40% by default. Users can change *τ* to keep more or less alignments.

## Results and discussion

We designed profile-based homology search method for NGS data lacking reference genomes, including RNA-Seq data of non-model species and metagenomic data. In order to demonstrate its utility in different types of data, we applied Short-Pair to a RNA-Seq dataset and a metagenomic dataset. In both experiments, we choose datasets with known reference genomes so that we can quantify the performance of homology search. It is important to note that the **ground truth** in this work is defined as the homology search results for complete genes. We are aware that computational protein domain annotation for complete genes or genomes are not always accurate. But whole-gene domain annotation has significantly higher sensitivity and accuracy than short read homology search and has been extensively tested in various species. Thus, our goal is to decrease the performance gap between short read homology search and whole-gene homology search.

HMMER can be run in different modes. In this work, we choose the most commonly used modes: HMMER with default E-value, HMMER with gathering thresholds (GAs) cutoff, and HMMER without filtration. GA cutoff is the recommended cutoff because of its accuracy. Turning off filtration will yield the highest sensitivity with sacrifice of speed.

The first dataset in our experiment is the RNA-Seq dataset of *Arabidopsis Thaliana*. The second one is metagenomic dataset sequenced from *bacterial* and *archaeal* synthetic communities. We will first carefully examine whether Short-Pair and HMMER can correctly assign each read to its correct domain families. Then we will evaluate the performance of homology search from users’ perspective. A user needs to know the composition of domains and also their abundance in a dataset. Thus we will compare HMMER and Short-Pair in both aspects.

### Profile-based short read homology search in *Arabidopsis Thaliana* RNA-Seq dataset

The RNA-Seq dataset was sequenced from a normalized cDNA library of *Arabidopsis* using paired-end sequencing of Illumina platform [21, 26]. There were 9,559,784 paired-end reads in total and the length of each read is 76 bp. The authors [26] indicated that the fragment lengths are between 198 and 801 bps. However, the fragment size distribution is unknown.

#### Determining the true membership of paired-end reads

The true membership of the short reads against protein families cannot be directly obtained by aligning the reads against protein families because of the low sensitivity and accuracy of short read alignment. The true membership was determined using read mapping and domain annotation on complete coding sequences. First, all coding sequences (CDS) of *Arabidopsis Thaliana* genome were downloaded from TAIR10 [27]. Second, we downloaded 3912 plant-related protein or domain models from Pfam [9]. We notice that some of these domain families are trained on genes of Arabidopsis. Thus, in order to conduct a fair evaluation of homology search performance, we removed all genes of *Arabidopsis* from the domain seed families and re-trained the Pfam profile HMMs. Third, CDS were aligned against Pfam domains [9] using HMMER with gathering thresholds (GAs) [1]. The alignment results contain the positions of domains in CDS. Note that it is possible that several domains are partially aligned to the same region in a coding sequence. This happens often for domains in the same clan [28] because these domains are related in structures and functions. In this case, we will keep all domain alignments passing the GA cutoff in the ground truth. Fourth, paired-end reads were mapped separately to CDS using Bowtie allowing up to 2 mismatches [29]. The positions of uniquely mapped reads in CDS were compared to annotated domains in CDS. If the mapping positions of read pairs are within annotated domain regions, we assigned the reads to those Pfam domains. The reads and their assigned domains constitute the true membership of these reads.

#### Performance of fragment length distribution

**Fig. 4:**
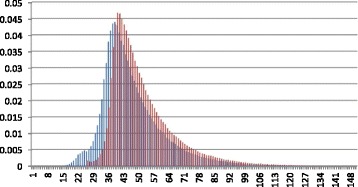
Comparing fragment length distribution of Short-Pair (blue) to fragment length distribution constructed from read mapping results (red) for *Arabidopsis* RNA-Seq dataset. X-axis represents the length of fragment in **amino acids**. Y-axis represents probability of the corresponding fragment size

We compared our estimated fragment length distribution with the true fragment length distribution in Fig. 4. The true fragment size distribution is derived by mapping all paired-end reads back to the reference genome. The comparison shows that, for a given length, the maximum probability difference between our fragment length distribution and the true fragment length distribution is 0.02, which slightly decreases the accuracy of the posterior probability calculation. It is worth nothing that in our experiments, we strictly removed all genes in the NGS data from the training sequences of the protein families/domains to create the case of no reference gene/sequence. In real applications, users can always try conducting read mapping first because some reference genes or genomes may exist in the public databases. The read mapping results, if available, can be used together with model-based fragment size estimation for generating more accurate size distribution.

#### Short-Pair can align significantly more reads

We applied HMMER and Short-Pair to annotate protein domains in this RNA-Seq dat set. Their alignments can be divided into three cases. Case 1: only one end can be aligned. Case 2: both ends can be aligned to the corresponding protein family. Case 3: neither end can be aligned. Case 2 is the ideal case. The results of this experiment were shown in Table 1. HMMER missed one end of at least half of the read pairs in the RNA-Seq dataset.Turning off filtration does not improve the percentage of case 2 substantially. Using gathering thresholds (GA) cutoff is recommended for accurate domain annotation in genomes. However, near 70% of read pairs cannot be aligned under GA cutoff. By applying Short-Pair, the percentage of case 2 (both ends) of paired-end read alignments increases from 28.42% to 62.51%. Importantly, the improvement is not achieved by sacrificing specificity. As we use the posterior probability to discard false alignments, the tradeoff between sensitivity and specificity is actually improved, as shown in the next section.

**Table 1:** The percentages of all three cases of paired-end read alignments by HMMER and Short-Pair for the *Arabidopsis* RNA-Seq dataset

Case	HMMER,	HMMER,	HMMER,	Short-Pair
	*E*-value 10	w/o filtration,	GA cutoff	
		*E*-value 10		
Case 1	34.51%	32.83%	22.51%	0.42%
Case 2	28.42%	31.58%	8.84%	62.51%
Case 3	37.07%	35.59%	68.65%	37.07%

“HMMER w/o filtration” : running HMMER by turning off all filtration steps. “HMMER GA cutoff”: applying HMMER with gathering thresholds

#### Sensitivity and accuracy of short read homology search

Although GA cutoff is the recommended threshold for domain annotation by HMMER, it yields low sensitivity for short read homology search. In order to align as many reads as possible, the default E-value cutoff is chosen. However, even for case 2, where both ends can be aligned by HMMER, these reads may be aligned to multiple domains by HMMER and not all of them are correct. Short-Pair can be used to improve the tradeoff between sensitivity and accuracy for both case 1 and case 2.

In this section, the performance of profile-based homology search for each read is quantified by comparing its true protein domain family membership and predicted membership. For each read pair, suppose it is sequenced from domain set *T* *P*={*T* *P*_1_,*T* *P*_2_,…,*T* *P*_*n*_}, which is derived from the read mapping results. The homology search tool aligns this read pair to domain set *C*={*C*_1_,*C*_2_,…,*C*_*m*_}. The sensitivity and false positive (FP) rate for this read pair are defined using the following equations:

2 $$ Sensitivity = \frac{\left|TP \cap C\right|}{\left|TP\right|}  $$

3 $$ FP\ rate = \frac{\left|C - TP\right|}{\left|TN\right|}  $$

Note that TN represents the true negative domain set. Let $\mathcal {U}$ represent all domains we downloaded from Pfam (|*U*|=3962). Then, for each read pair, *T* *N*=*U*−*T* *P*. In this section, the sensitivity and FP rate for each pair of reads are computed and then the average of all pairs of reads is reported using ROC curves.

##### Performance of case 1:

**Fig. 5:**
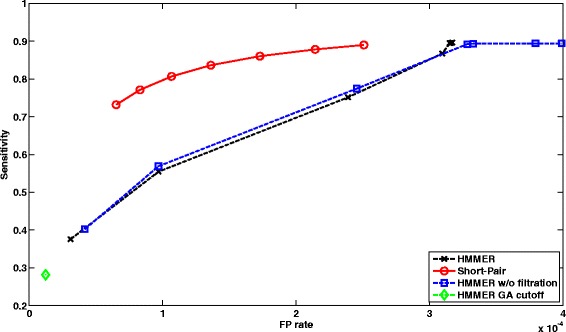
ROC curves of profile-based short read homology search for *Arabidopsis* RNA-Seq data. We compared HMMER and Short-Pair on case 1, where one end can be aligned by HMMER with default E-value. Note that HMMER with GA cutoff has one data point

There are 1,025,982 paired-end reads, where only one end can be aligned to one or multiple domain families by HMMER with filtration on. Figure 5 shows ROC curves of short read homology search using HMMER under different cutoffs and Short-Pair. For HMMER, we changed the E-value cutoff from 1000 to 10^−5^ with ratio 0.1. As some E-value cutoffs yield the same output, several data points overlap completely. For Short-Pair, each data point corresponds to different *τ* values (10 to 70%) as defined in “Probabilistic model” Section. Unless specified otherwise, all the ROC curves are generated using the same configuration.

##### Performance of case 2:

**Fig. 6:**
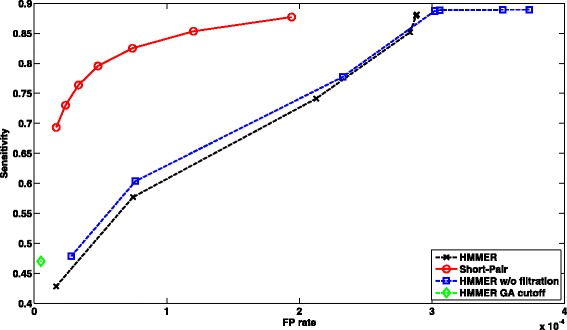
ROC curves of profile-based short read homology search for *Arabidopsis* RNA-Seq data. We compared HMMER and Short-Pair on case 2, where both ends are aligned by HMMER with default E-value. Note that HMMER with GA cutoff has one data point. Using posterior probability helps remove false aligned domains and thus leads to better tradeoff between sensitivity and FP rate

There are 844,796 paired-end reads with both ends being aligned by HMMER with filtration on. Some read pairs are aligned to false families. The falsely aligned domain families can be removed by Short-Pair. Therefore, Short-Pair have better trade-off between sensitivity and false positive rate. In Fig. 6, we plotted ROC curves of HMMER and Short-Pair.

The results showed that HMMER with GA cutoff yields low sensitivity and low FP rate. Short-Pair has better tradeoff between sensitivity and FP rate for both cases. We also computed other metrics including F-score $\left (\frac {2 \times sensitivity \times PPV}{sensitivity + PPV}\right)$ and PPV $\left (\text {Positive Predictive Value,} \frac {\left |TP \cap C\right |}{\left |C\right |}\right)$. Comparing all tools in terms of F-Score and PPV under different thresholds for case 1, Short-Pair achieves the highest F-Score 81.98%; the corresponding PPV is 80.41%. HMMER w/o filtration has the second highest F-Score 75.39% and its PPV is 65.17%. For case 2, Short-Pair has the highest F-score 86.33% with PPV 94.34%. HMMER with default E-value cutoff has the second highest F-Score 76.45% with PPV 67.50%.

#### Performance evaluation on domain-level

In order to assess the homology search performance on domain-level, we focused on comparing the set of domains found by HMMER and Short-Pair. We further quantified the domain abundance, which is the number of reads classified in each domain by given tools. The predicted domain set and their abundance are also compared to the ground truth, which is derived using the read mapping results and the whole-gene domain annotation.

Our experimental results showed that the set of domains reported by HMMER under the default E-value cutoff and Short-Pair are almost identical. They only differ by 1 out of 3962 domains. Both tools can identify almost all the ground-truth domains. The only exception is HMMER with GA cutoff, which returns 84% of true domains.

**Fig. 7:**
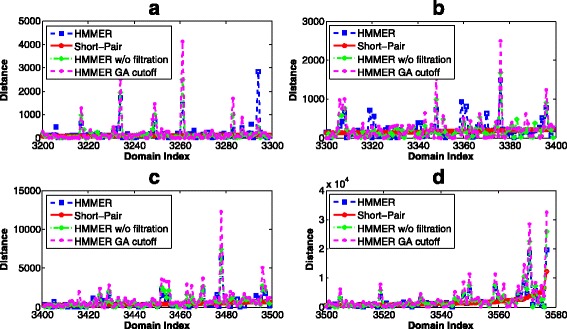
The distance comparison between Short-Pair and HMMER on case 1 of the RNA-Seq dataset of *Arabidopsis*. 377 domains with the largest distance values starting from domain index 3201 to domain index 3577 are listed in the four subplots: **a**, **b**, **c**, and **d**. X-axis shows the indices of the domains. Smaller value indicates closer domain abundance to the ground truth. The average distances of HMMER, HMMER w/o filtration, HMMER GA cutoff, and Short-Pair are 704.92, 781.80, 1,054.77, and 522.12, respectively

Although HMMER and Short-Pair reported near identical domain sets, they generated very different domain abundance. We compared the predicted abundance to the ground truth by computing their distance, which is the difference of the number of reads classified to a domain. According to the definition, small distance indicates higher similarity to the ground truth. For case 1, Short-Pair has smaller distance to the ground truth than HMMER, with average distance being 65.39. Short-Pair produced the same abundance as the ground truth for 1,185 domains. The average distances of HMMER, HMMER without filtration, and HMMER with GA cutoff are 107.60, 126.85, and 153.64, respectively. Figure 7 shows the distance of 377 domains for which Short-Pair has distance above 86.

**Fig. 8:**
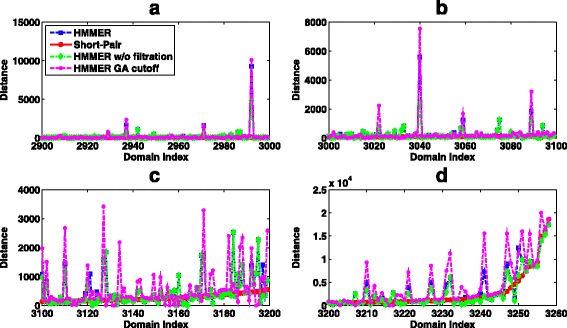
The distance comparison between Short-Pair and HMMER on case 2 of the RNA-Seq dataset of *Arabidopsis*. Three hundred fifty eight domains (Domain index: 2901 - 3258) with the largest distances are listed in the four subplots: **a**, **b**, **c**, and **d**. X-axis shows the indices of the domains. Smaller value indicates closer domain abundance to the ground truth. The average distances of HMMER, HMMER w/o filtration, HMMER GA cutoff, and Short-Pair are 818.09, 704.65, 1084.50, and 558.60, respectively

Figure 8 illustrates the distance between the predicted domain abundance and the ground truth for case 2, where both ends can be aligned by HMMER under the default E-value cutoff. The average distances for HMMER, HMMER without filtration, HMMER with GA cutoff, and Short-Pair are 121.61, 107.81, 139.56, and 96.34 respectively. Figure 8 only includes 358 domains for which Short-Pair has the distance above 30.

In summary, being consistent with the results shown in Figs. 5 and 6, Short-Pair can assign reads to their native domains with higher accuracy.

#### Running time analysis

We compared the running time of tested tools in Table 2. HMMER with GA cutoff is the fastest but yields low sensitivity. HMMER without filtration is computationally expensive and is the slowest. We are in between as we rely on the full Viterbi algorithm to align the missing end of a read pair.

**Table 2:** The running time of HMMER under different cutoffs and Short-Pair on the *Arabidopsis Thaliana* RNA-Seq dataset

Case	HMMER,	HMMER,	HMMER,	Short-Pair
	*E*-value 10	w/o filtration,	GA cutoff	
		*E*-value 10		
Time (m)	0.66	191.53	0.51	2.48

m: minutes. *Note:* The running time is the average running time of aligning 9,559,784 paired-end reads with a domain

### Profile homology search for short reads in a metagenomic dataset from synthetic communities

In the second experiment, we tested the performance of short read homology search in a metagenomic dataset. In order to quantify the performance of Short-Pair, we chose a mock metagenomic data with known composition.

#### Dataset

The chosen metagenomic data set is sequenced from diverse synthetic communities of *Archaea* and *Bacteria*. The synthetic communities consist of 16 *Archaea* and 48 *Bacteria* [30]. All known genomes were downloaded from NCBI. The metagenomic dataset of synthetic communities were downloaded from NCBI Sequence Read Archive (SRA) (accession No. SRA059004). There are 52,486,341 paired-end reads in total and the length of each read is 101 bp. All of reads are aligned against a set of single copy genes. These genes includes nearly all ribosomal proteins as well as tRNA synthases existed in nearly all free-living bacteria [31]. These protein families have been used for phylogenetic analysis in various metagenomic studies and thus it is important to study their composition and abundance in various metagenomic data. We downloaded 111 domains from Pfam database [9] and TIGRFAMs [11].

#### Determination of true membership of paired-end reads

The true membership of paired-end reads is determined based on whole coding sequence annotation and read mapping results. First, all coding sequences (CDS) of 64 genomes of *Archaea* and *Bacteria* were downloaded from NCBI. Second, CDS were aligned against 111 domains downloaded from TIGRFAMs [11] and Pfam database [9] using HMMER with gathering thresholds (GAs) [1]. The positions of aligned domains in all in CDS were recorded. Third, paired-end reads were mapped back to the genomes using Bowtie [29]. The read mapping positions and the annotated domain positions are compared. If both ends are uniquely mapped within an annotated domain, we assign the read pair to the domain family. The true positive set contains all read pairs with both ends being uniquely mapped to a protein domain. We will only evaluate the homology search performance of chosen tools for these reads.

#### Performance of fragment length distribution

**Fig. 9:**
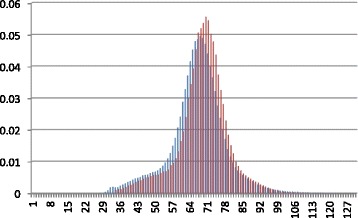
Comparing fragment length distribution of Short-Pair (blue) to fragment length distribution constructed from read mapping results (red) for the synthetic metagenomic dataset. X-axis represents fragment length in **amino acids**. Y-axis represents the probability of the corresponding fragment size

Again, we need to examine the accuracy of our fragment size computation. Figure 9 shows the fragment length distribution constructed from Short-Pair and the fragment length distribution derived from the read mapping results. For a given length, the maximum probability difference between Short-Pair and the ground truth is 0.01, which slightly reduces the accuracy of posterior probability computation.

#### Short-Pair can align more reads

In this experiment, the read length is longer than those in the first experiment. Consequently, HMMER can align more reads against their native domain families. Nevertheless, it still has one third of pairs of reads with one end being aligned to the protein domain families. By applying Short-Pair, the percentage of case 2 (both ends) of paired-end read alignments is enhanced from 65.82% to 88.71%. The percentages of three cases by Short-Pair and HMMER are shown in Table 3.

**Table 3:** The percentages of all three cases of paired-end read alignments by HMMER and Short-Pair for the synthetic metagenomic dataset

Case	HMMER,	HMMER,	HMMER,	Short-Pair
	*E*-value 10	w/o filtration,	GA cutoff	
		*E*-value 10		
Case 1	23.15%	21.63%	3.76%	0.26%
Case 2	65.82%	68.46%	2.46%	88.71%
Case 3	11.03%	9.91%	93.77%	11.03%

Case 1: only one end aligned. Case 2: both ends aligned. Case 3: no end aligned

#### Sensitivity and accuracy of short read homology search

##### Case 1: one end is aligned by HMMER

**Fig. 10:**
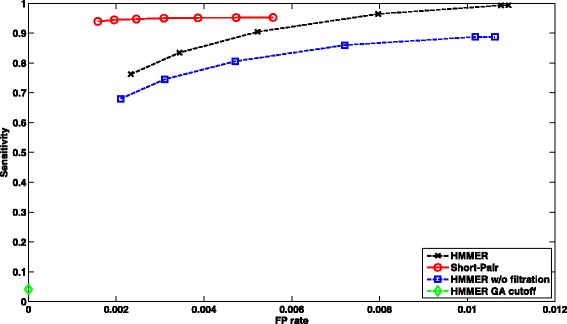
ROC curves of profile-based short read homology search for the synthetic metagenomic dataset. We compared HMMER and Short-Pair on case 1, where one end can be aligned by HMMER with default E-value. Note that HMMER under GA cutoff has one data point

There were 213,668 paired-end reads with only one end being aligned to one or multiple domains. Figure 10 shows the ROC curves of short read homology search using HMMER and Short-Pair. HMMER with GA cutoff has the lowest FP rate (0.0). However, the sensitivity of HMMER with GA cutoff is only 4.11%. In addition, we further computed PPV and F-Score of each data point in ROC curves. Comparing all tools, Short-Pair has the highest F-Score and PPV (90.87% and 88.01%, respectively). HMMER with E-value 10 has the next highest F-score and PPV (64.79% and 48.07%, respectively).

##### Case 2: both ends are aligned by HMMER

**Fig. 11:**
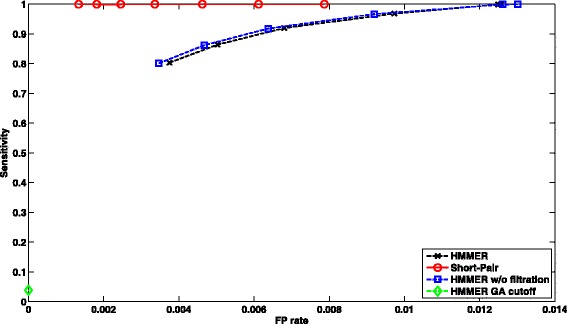
ROC curves of profile-based short read homology search for the synthetic metagenomic dataset. We compared HMMER and Short-Pair on case 2, where both ends are aligned by HMMER under default E-value. Note that HMMER under GA cutoff has one data point. Using posterior probability helps remove false aligned domains and thus leads to better tradeoff between sensitivity and FP rate

607,558 paired-end reads were classified to case 2. We divided data into two groups: 1) both ends being aligned to one domain and 2) both ends being aligned to multiple domains. There were 515,586 paired-end reads and 91,972 paired-end reads, respectively. When both ends are aligned to one single domain, the classification is usually correct. Thus, we focus on evaluating the performance of the second group, where read pairs are aligned to more than one domain. Figure 11 shows the average performance comparison between HMMER and Short-Pair on 91,972 paired-end reads. Comparing all tools in term of F-Score and PPV, Short-Pair achieves the highest F-Score of 96.05% and its PPV is 92.42%. HMMER w/o filtration achieves the second highest F-Score 80.28% with PPV 80.45%.

#### Domain-level performance evaluation

For whole dataset, we compared the set of domains identified by HMMER and Short-Pair. The results showed that every tool identified all ground truth domains (111 domains) except HMMER with GA cutoff, which only found 26 domains.

**Fig. 12:**
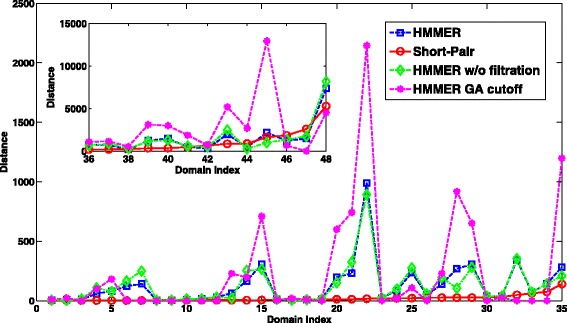
The distance comparison between Short-Pair and HMMER on case 1 of the metagenomic dataset. X-axis shows the indices of the domains. Smaller value indicates closer domain abundance to the ground truth. Domains are sorted based on the distance of Short-Pair. Due to scaling issues, domains with the largest distances are plotted in the embedded window

In addition, the domain abundance was quantified and compared to the ground truth. For each domain, we compute the “distance”, which is the difference in the number of reads classified to a domain by a tool and in the ground truth. Smaller distance indicates closer domain abundance to the ground truth. For case 1, the average distances of HMMER, HMMER w/o filtration, HMMER with GA cutoff, and Short-Pair are 272.74, 280.65, 505.56, and 178.60, respectively. Short-Pair has the same abundance as the ground truth in 43 domains. We removed those 43 domains and showed distance of other domains in Fig. 12.

**Fig. 13:**
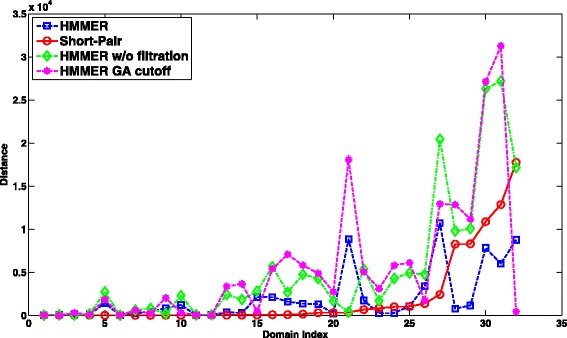
The distance comparison between Short-Pair and HMMER on case 2 of the metagenomic dataset. X-axis shows the indices of domains. Smaller value indicates closer domain abundance to the ground truth

For case 2, where both ends can be aligned, all tools have worse domain abundance estimation. The average distances of HMMER, HMMER w/o filtration, HMMER with GA cutoff, and Short-Pair are 702.39, 1698.79, 1831.55, and 666.96, respectively. Short-Pair still has the closest domain abundance to the ground truth. It has the same domain abundance as the ground truth for 68 domains. We removed the 68 domains and plotted the distances of other domains in Fig. 13.

Although the read lengths of this data set are longer than the first data set, the average sequence conservation of the domain families is as low as 30%. The poorly conserved families contain large numbers of substitutions, long insertions and deletions, leading to either over-prediction or under-prediction of the tested tools. HMMER with E-value cutoff 10, HMMER w/o filtration, and Short-Pair all classified significantly more reads into the domain families than ground truth. HMMER with GA cutoff significantly under-classified short reads into the underlying families. Thus, the distances of all these tools are large.

#### Running time on a metagenomic dataset of synthetic communities

The running times of HMMER under different cutoffs and Short-Pair are compared in Table 4. As expected, HMMER with filtration is the fastest. Short-Pair is slower than HMMER with filtration but much faster than HMMER w/o filtration.

**Table 4:** The running time of HMMER under different cutoffs and Short-Pair on a metagenomic dataset of synthetic communities

Case	HMMER,	HMMER,	HMMER,	Short-Pair
	*E*-value 10	w/o filtration,	GA cutoff	
		*E*-value 10		
Time (m)	3.18	1,377.15	3.41	40.25

m: minutes. *Note:* The running time is the average running time of aligning 52,486,341 paired-end reads against one domain family

## Conclusion

Homology search has been widely used for sequence-based functional analysis in various NGS sequencing projects. In particular, for gene-centric analysis, reads are classified into characterized protein/domain families using profile-based homology search. While HMMER is the state-of-the-art tool for profile homology search, its performance on short reads has not been systematically examined. Our test of HMMER in various NGS data containing short reads shows that it could miss a large number of short reads. In this work, we described a probabilistic homology search model for paired-end reads. The goal is to improve the performance of short read homology search. It is built on HMMER and can be used as a complementary tool to HMMER for more sensitive read classification.

One future direction is to improve the short read homology search performance for poorly conserved families. Near 4000 domain families in the first experiment have higher average sequence identity and thus lead to reasonable domain abundance estimation. The 100+ families in the second experiment have low sequence identity and the tested tools tend to either over-classify or under-classify heavily for some families. Thus, better methods need to be designed to align short reads to poorly conserved protein families.

The advances of NGS technologies enable output of longer reads. The increased length will lead to better sensitivity of HMMER. However, before the reads reach the length of near complete transcripts or genes, there is still a need for improving short read homology search. In addition, existing sequencing projects are still heavily relying on today’s sequencing technologies. We expect Short-Pair can be used to improve the functional analysis.
